# An RBFNN-Based Prescribed Performance Controller for Spacecraft Proximity Operations with Collision Avoidance

**DOI:** 10.3390/s26010108

**Published:** 2025-12-23

**Authors:** Xianghua Xie, Weidong Chen, Chengkai Xia, Jiajian Xing, Liang Chang

**Affiliations:** 1 State Key Laboratory of Mechanics and Control for Aerospace Structures, Nanjing University of Aeronautics and Astronautics, Nanjing 210016, China; 2 Innovation Academy for Microsatellites of Chinese Academy of Science, Shanghai 201203, China

**Keywords:** spacecraft, motion control, predefined boundary, adaptive neural control

## Abstract

In the mission scenario of On-Orbit Assembly (OOA), servicing spacecraft are frequently tasked with towing large-scale, flexible truss structures to designated assembly sites. This process involves complex coupled dynamics between the spacecraft and the flexible payload, which are often unmodeled or unknown, posing significant challenges to control precision. Furthermore, the proximity of other assembled structures in the construction area necessitates strict collision avoidance. To address these challenges, this paper proposes a novel adaptive robust controller for spacecraft thruster-based orbital control that integrates Prescribed Performance Control (PPC) with a Radial Basis Function Neural Network (RBFNN). The PPC framework ensures that the position tracking errors remain within user-predefined, time-varying boundaries, providing an intrinsic mechanism for collision avoidance during the towing of large flexible structures. Concurrently, the RBFNN is employed to approximate the entire unknown nonlinear dynamics of the combined spacecraft-truss system online, effectively compensating for uncertainties arising from the flexibility of the truss and external disturbances. The performance of the proposed controller is validated through both numerical simulations and hardware experiments on a ground-based air-bearing satellite simulator. Simulation results demonstrate the controller’s superior tracking accuracy compared to a conventional PID controller, while strictly adhering to the prescribed error constraints. Experimental results further confirm its effectiveness, showing that the simulator can track a desired trajectory with high precision, with tracking errors converging to approximately 5 mm while consistently remaining within the predefined safety boundaries. The proposed approach provides a robust and safe control solution for complex proximity operations in on-orbit construction, eliminating the need for precise dynamic modeling of flexible payloads.

## 1. Introduction

With the rapid advancement of space technology, On-Orbit Assembly (OOA) of large-scale space infrastructures—such as space solar power stations and large-aperture space telescopes—has become a critical frontier in aerospace engineering. In these missions, servicing spacecraft are often required to act as “space tugs,” utilizing adhesion mechanisms or robotic arms to tow large truss sub-assemblies from a delivery orbit to a designated construction site for assembly [1]. This specific operational scenario imposes stringent requirements on the precision, safety, and robustness of the control system. The successful execution of such on-orbit construction and material interaction tasks relies heavily on addressing the dual challenges of manipulating large flexible payloads and ensuring safe proximity operations in cluttered environments.

Firstly, the dynamics of the combined system—comprising the servicing spacecraft and the towed truss—are highly complex and uncertain. The truss structures used in OOA are typically characterized by large dimensions, low stiffness, and significant flexibility [2,3,4]. During the towing process, the excitation of flexible vibration modes in the truss can introduce severe disturbances to the servicing spacecraft. Furthermore, the mass, inertia tensor, and damping characteristics of the towed object are often unknown or time-varying, rendering it difficult to establish a precise mathematical model for the combined system [5]. Traditional model-based control methods, which rely on accurate dynamic parameters, often fail to maintain stability and tracking performance under such significant uncertainties.

Secondly, safety—specifically collision avoidance—is the paramount concern in the assembly zone. The servicing spacecraft must maneuver the large truss to the target location, which is often in close proximity to other partially or fully assembled structures [6,7]. Unlike open-space rendezvous, the “construction site” environment is constrained, where any deviation beyond a safe corridor could lead to a collision between the towed truss and the existing infrastructure, potentially causing catastrophic mission failure. Therefore, the control system must possess the capability to actively constrain the trajectory error within a predefined safe envelope throughout the entire towing maneuver. This necessitates a control strategy that offers explicit guarantees on transient and steady-state tracking errors, rather than merely asymptotic convergence.

**Figure 1:**
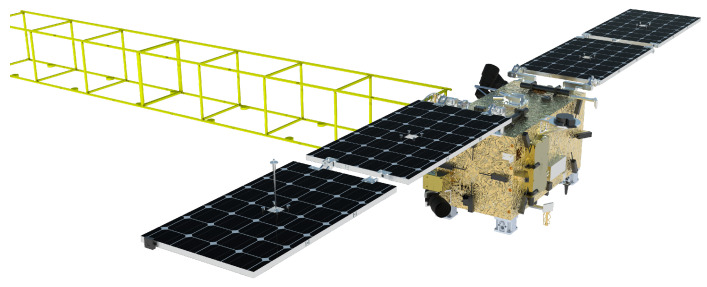
Conceptual illustration of the on-orbit towing scenario.

The operational scenario targeted in this research is depicted in Figure 1. A servicing spacecraft utilizes an adhesion mechanism to tow a long truss segment. The mission requires the spacecraft to transport the truss to a specific assembly point where specialized robots will integrate it into a larger facility. The vicinity of the target location may contain other trusses, mandating strict collision avoidance. Furthermore, the trusses vary in specifications (length, mass, attached subsystems), requiring the spacecraft to adaptively control the motion without prior knowledge of the specific truss parameters.

Traditional control methods often reveal their inherent limitations when addressing the complex demands of spacecraft proximity operations. Proportional–Integral–Derivative (PID) controllers, widely used due to their simple structure and ease of implementation, often exhibit unsatisfactory performance when dealing with highly nonlinear, strongly coupled complex dynamic systems like servicing spacecraft [8]. PID controller parameters are difficult to tune, making it challenging to balance responsiveness with overshoot and steady-state accuracy, especially when system parameters are time-varying or unmodeled dynamics exist; their control precision and convergence speed significantly degrade. More importantly, PID controllers are essentially error feedback controllers, lacking the explicit ability to constrain error boundaries, thus failing to fundamentally guarantee collision safety during complex dynamic processes. On the other hand, model-based control methods, such as computed torque control or feedback linearization, while theoretically capable of achieving high-performance control for nonlinear systems, heavily rely on accurate system dynamic models [9]. In space applications, obtaining an accurate dynamic model is extremely difficult, particularly when the robot is interacting with a target whose properties are unknown or change dynamically. Unmodeled dynamics, parameter perturbations (e.g., payload changes, fuel consumption), and persistent external environmental disturbances all lead to model mismatch, thereby significantly weakening the performance and robustness of such controllers [2,5]. The peculiarities of the space environment, such as microgravity, vacuum, strong radiation, and communication limitations, further exacerbate the shortcomings of these traditional control methods, highlighting the urgent need for developing novel advanced control strategies [2,10].

To overcome the deficiencies of traditional control methods and meet the pressing demands for high precision, high safety, and high robustness in spacecraft proximity operations, researchers have begun to explore more advanced control theories. For instance, optimized backstepping control has been successfully applied to attitude containment for multiple spacecrafts, offering effective solutions for cooperative maneuvering [11,12,13]. Regarding collision avoidance strategies, Artificial Potential Functions (APFs) represent a foundational and widely adopted methodology [14,15]. APF-based methods construct a virtual potential field where obstacles generate repulsive forces and the target generates attractive forces, enabling effective path planning and obstacle avoidance [16,17], while APFs are highly effective and computationally efficient, they may suffer from inherent limitations such as local minima traps, which can lead to system stagnation or oscillatory behavior near obstacles. Furthermore, standard APFs typically act as “soft” constraints via force generation, which might not strictly guarantee safety boundaries under extreme disturbances. In contrast, PPC, also known as prescribed error boundary control, offers a distinct advantage for the mission scenario considered in this paper [18,19]. By transforming the tracking error into a constrained space, PPC imposes “hard” constraints on the system’s trajectory. PPC predefines a performance function to constrain the system’s tracking error, ensuring that the error remains within preset time-varying boundaries throughout the control process and eventually converges to a sufficiently small residual set. This method not only guarantees the system’s transient and steady-state performance (such as convergence speed and overshoot), but, more importantly, by appropriately setting the error boundaries, it can directly serve safety objectives like collision avoidance [3,20,21,22]. However, the effectiveness of PPC still relies on some understanding of the system’s dynamic characteristics. To cope with the prevalent model uncertainties and external disturbances in spacecraft systems, RBFNNs have garnered significant attention due to their powerful nonlinear approximation capabilities and online learning characteristics [23,24]. In the context of this research, the RBFNN essentially functions as a “soft sensor” or online estimator. Since physical sensors cannot directly measure the complex aggregated disturbances (including flexible vibrations and mass variations), the RBFNN identifies these unmeasurable dynamic states from the available position and velocity data. RBFNNs can approximate any continuous function with arbitrary precision, and their relatively simple structure and fast training speed make them highly suitable for online identification and compensation of unknown system dynamics or external disturbances [3,4,25]. Combining PPC with RBFNNs is expected to leverage their complementary advantages: PPC provides a strict performance guarantee and safety framework, while RBFNNs, through online learning, compensate for the system’s uncertain dynamics in real-time, thereby achieving high-precision, high-safety autonomous proximity control in complex and variable space environments. The core idea of this paper is based on this, proposing a novel controller where an RBFNN directly fits the system’s dynamic model online and is integrated into the PPC framework.

The main contributions of this paper lie in the design and analysis of a novel RBFNN-based prescribed-boundary controller, specifically for thruster control of servicing spacecraft during proximity operations such as approach, station-keeping, and grappling. Specifically, the controller proposed in this paper features the following:

- High-precision trajectory tracking and station-keeping control: Achieves high-precision tracking of desired trajectories and stable station-keeping near the target through the online adaptive learning of RBFNN and the strict error constraints of PPC [3,4].
- Collision avoidance based on prescribed error boundaries: The application of PPC ensures that relative position and attitude errors always remain within user-predefined time-varying boundaries, thus providing intrinsic safety assurance for close-range operations and effectively avoiding collisions with the target [18,22].
- RBF neural network online approximation of system dynamics: Unlike traditional methods that only use RBFNN for disturbance compensation, this paper employs RBFNN to directly identify and fit the complex, uncertain nonlinear dynamic model of the spacecraft online, thereby more effectively addressing significant changes in model parameters and unmodeled dynamics [23,25].
- Strong robustness against model uncertainties and external disturbances: The synergistic effect of the PPC framework and RBFNN dynamic compensation endows the controller with strong robustness against inherent parameter uncertainties in the spacecraft system (such as mass and inertia changes after target capture) and persistent external disturbances in the space environment (such as gravity gradients, solar radiation pressure, etc.) [2,26].

The remainder of this paper is organized as follows: Section 2 details the design of the spacecraft motion control system and its simulator, including coordinate system definitions, the orbital dynamics model of the spacecraft, motion control system design, and ground simulator design. Section 3 focuses on the design principles and implementation details of the RBFNN-based prescribed-boundary control algorithm. Section 4 validates the performance of the proposed control algorithm through simulation experiments under various conditions. Section 5 analyzes the stability of the control algorithm. Section 6 presents the experimental verification results based on the ground simulator. Section 7 concludes the entire work and discusses future research directions.

## 2. Spacecraft Motion Control System and Its Simulator Design

### 2.1. Definition of Spacecraft Coordinate Systems

**Figure 2:**
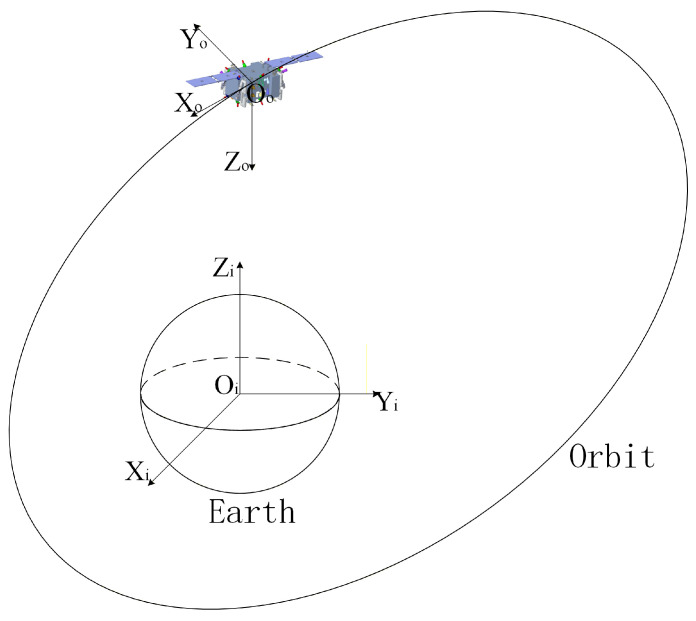
Schematic diagram of inertial and orbital coordinate systems.

**Figure 3:**
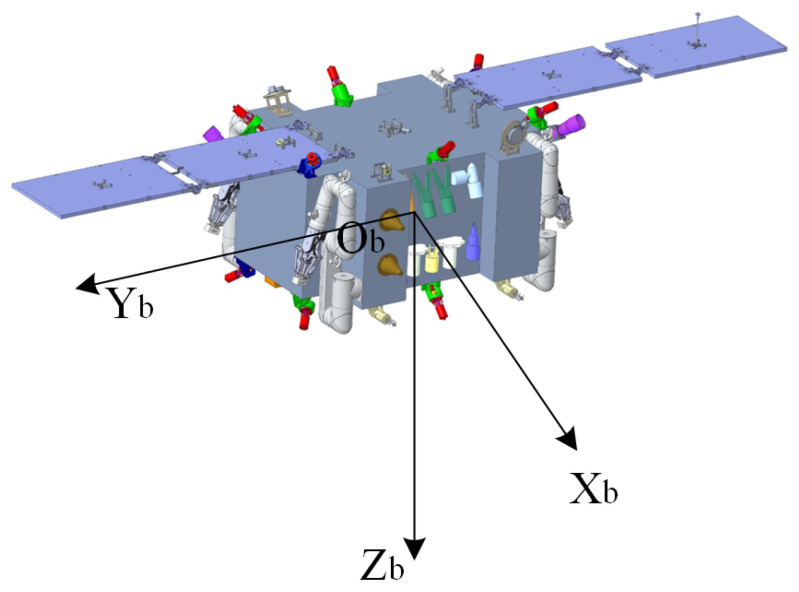
Schematic diagram of the spacecraft body-fixed coordinate system.

The motion description of a spacecraft is based on precise relative kinematic equations, and the establishment of relative motion equations requires unified coordinate systems. The following are definitions of several coordinate systems used in this paper, as shown in Figure 2 and Figure 3.

- Inertial Coordinate System $F_{i}$: $O_{i}X_{i}Y_{i}Z_{i}$ is the Geocentric Equatorial Inertial (GEI) coordinate system. Its origin is at the Earth’s center, the $OX_{i}$ axis points towards the vernal equinox in the equatorial plane, the $OZ_{i}$ axis is perpendicular to the equatorial plane pointing towards the North Pole along the normal, and the $OY_{i}$ axis is determined by the right-hand rule.
- Orbital Coordinate System $F_{o}$: $OX_{o}Y_{o}Z_{o}$ is the center-of-mass orbital coordinate system. Its origin is at the spacecraft’s center of mass, $OZ_{o}$ points towards the Earth’s center, the $OX_{o}$ axis is in the orbital plane, perpendicular to the $OZ_{o}$ axis, and points in the direction of the spacecraft’s motion. The $OY_{o}$ axis forms a right-handed orthogonal coordinate system with the $OX_{o}$ and $OZ_{o}$ axes.
- Body-Fixed Coordinate System $F_{b}$: $OX_{b}Y_{b}Z_{b}$. Its origin is at the spacecraft’s center of mass. The $X_{b}$ axis points in the forward direction, called the roll axis; the $Y_{b}$ axis is perpendicular to the longitudinal plane of symmetry, called the pitch axis; the $Z_{b}$ axis forms a right-handed orthogonal coordinate system with the other two axes, called the yaw axis. If the spacecraft is not rotating, it coincides with the orbital coordinate system $OX_{o}Y_{o}Z_{o}$.

### 2.2. Spacecraft Motion Control System Design

The designed spacecraft must be capable of detecting and orbit determination of non-cooperative space targets (i.e., autonomous navigation) and precise identification of maintenance surfaces (i.e., image recognition). The spacecraft is required to have full 6-degree-of-freedom (DOF) control capability. To simplify the control system design, the attitude control and orbit control (i.e., conventional position control) of the spacecraft are separated into two independent control systems: the attitude control system and the orbit control system.

The attitude control system uses star trackers and fiber optic gyroscopes as attitude measurement inputs, and momentum wheels and thrusters (installed in pairs with opposing thrust directions) as actuators. Momentum wheels achieve small control torque output using the principle of momentum exchange. Since the momentum capacity of momentum wheels is limited and the designed spacecraft operates in low Earth orbit, magnetic torquers are designed to unload the momentum wheels during periods not requiring high-precision control.

**Figure 4:**
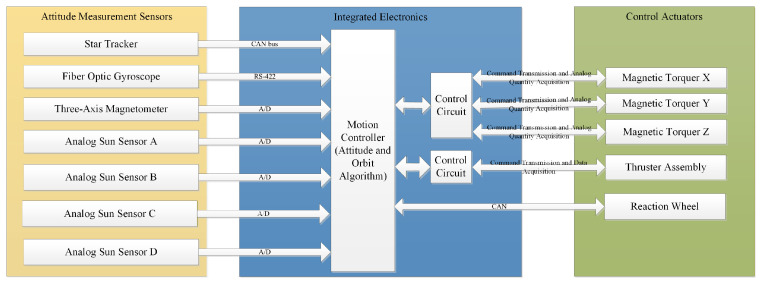
Schematic diagram of the attitude and orbit control system structure.

**Figure 5:**
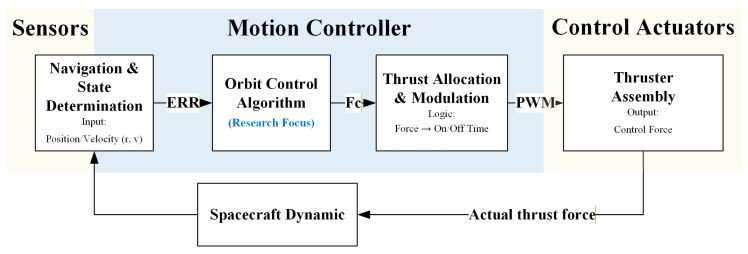
Logical data flow within the Motion Controller.

The orbit control system uses a laser radar camera and a Time-of-Flight (TOF) camera as mutual backups to measure the relative position with respect to the target spacecraft. The motion control system of the spacecraft can be divided into an attitude control system and an orbit control system. The attitude control system adopts an offset nadir-pointing scheme and a jet modulation control scheme. It uses star trackers and fiber optic gyroscopes as high-precision primary attitude determination components to meet mission requirements, and is also configured with differential sun sensors and magnetometers for coarse attitude determination or for attitude reacquisition in case of attitude loss. The specific composition is shown in Figure 4 and Figure 5.

It is noteworthy that the orbit control system employs a cold gas propulsion scheme. Micro n-butane propulsion modules are selected. Each thruster provides a thrust of 0.005 N. There are 12 thrusters for orbit control, installed in the ±X,±Y,±Z directions, with two thrusters per direction, achieving a combined force of 0.01N. The combined force of the thrusters in each direction passes through the spacecraft’s center of mass. The pair of thrusters in each direction is controlled using PWM to control the three-axis translation of the spacecraft.

### 2.3. Ground Simulator Design

The ground simulator includes a control subsystem, a gas and power supply subsystem, an intelligent recognition subsystem, and a comprehensive management subsystem. The control subsystem mainly includes the actuators on the ground simulator and their auxiliary devices. The power and gas supply subsystem is primarily responsible for the power and gas supply of the simulator and also includes the air bearing subsystem. The intelligent recognition subsystem is responsible for measuring the pose information of the simulator. The comprehensive management subsystem is mainly used for the upper-level control and status monitoring of the simulator.

**Figure 6:**
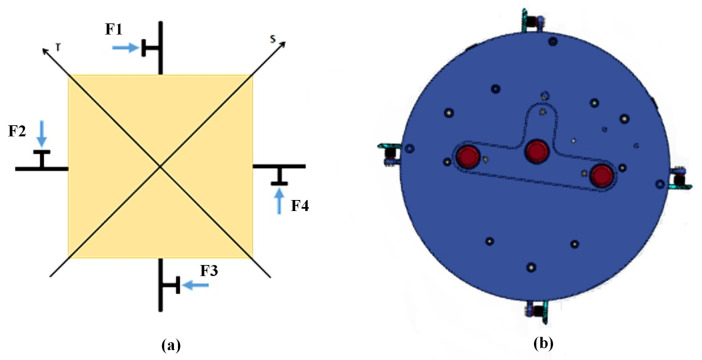
(**a**) Actuator installation schematic diagram; (**b**) Installation location of the actuator.

The control subsystem includes an instrument platform, a momentum exchange system, and a pose control system. The instrument platform is the mounting platform for the entire ground simulator. According to working conditions and external interface constraints, the instrument platform is divided into three layers: the lower layer is used for installing air bearings and the gas supply subsystem to facilitate the charging and discharging of gas storage cylinders, reduce redundant gas line layout, and lower the platform’s center of gravity; the middle layer is used for installing the momentum exchange system and pose control system; the upper layer is used for the marker lights of the intelligent recognition subsystem to meet the functional characteristics of the air flotation platform. The momentum exchange system, i.e., the actuators, consists of a servo fan group and flywheels arranged in an orthogonally symmetric manner. It achieves control of the ground simulator through airflow and momentum exchange, as shown in Figure 6. The servo fan group can control thrust magnitude by adjusting speed and direction. The pose control system consists of an industrial control computer and a driver board. The industrial control computer runs the control algorithm and transmits control commands to the driver board via an RS232 serial port, which then controls the momentum exchange system.

The power and gas supply system consists of three parts: air bearings, a gas source system, and a power system. The air bearing subsystem, i.e., air feet, allows high-pressure gas from the source to flow through throttling orifices in the air feet into the gap between the lower surface of the air feet and the high-precision granite platform, forming an air film. This generates an upward force, suspending the air flotation platform system mounted on the air feet, enabling the air flotation robot to achieve three-degree-of-freedom motion. Gravity interference is reduced by precisely adjusting the flatness of the platform. The gas source system, composed of high-pressure gas cylinders, high- and low-pressure gas lines, inflation switches, and pressure reducing valves, is responsible for providing stable air pressure and flow to the air bearings. The power system, consisting of a 10,000 mAh rechargeable lithium battery, a charger, and voltage stabilization and conversion modules, is responsible for providing stable voltage and current to each unit. The power system can continuously supply power for 1 h and 10 min when fully charged.

**Figure 7:**
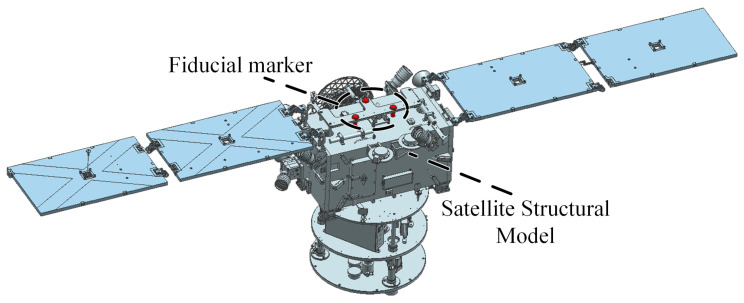
Design drawing of the ground simulator target.

The intelligent recognition subsystem is based on computer vision principles and uses a monocular camera measurement scheme, as shown in Figure 7, with the target on top of the air flotation robot as a reference, to achieve pose measurement of the ground simulator.

The comprehensive management subsystem includes a host computer system and a communication link with the pose control system. The host computer system is used to plan the motion trajectory and attitude of the ground simulator and receive the simulator’s pose measured by the intelligent recognition subsystem. It transmits the actual pose and target pose at each moment to the pose control system on the ground simulator via wireless transmission. The comprehensive management subsystem can also monitor the attitude information of the ground simulator in real-time.

## 3. Control Algorithm Design

During on-orbit construction tasks, the spacecraft is frequently required to tow large truss components. These components often possess significant flexibility and varying mass distributions, making it impractical to develop a precise dynamic model for every possible towing configuration. The flexibility of the truss introduces unmodeled vibrational modes that act as complex nonlinear disturbances to the servicing spacecraft. Furthermore, strict collision avoidance is mandatory during the approach and towing phases to protect the assembly site. To address these challenges, this paper adopts a control strategy that utilizes an RBFNN to approximate the unknown dynamics of the combined spacecraft-truss system, integrated with a PPC framework to ensure safety through rigorous error confinement.

The orbit control of the spacecraft (i.e., translational control) is designed such that the three translational degrees of freedom are decoupled. Therefore, the dynamics for the *j*-th axis (j∈{1,2,3}, corresponding to the X, Y, and Z axes) can be described as a second-order nonlinear system:

(1) $$\begin{array}{c} \left\{\begin{array}{ccc} {\dot{x}}_{1j}\left(t\right) & = & x_{2j}\left(t\right)\\ {\dot{x}}_{2j}\left(t\right) & = & \varphi_{j}\left(x_{j}\right)u_{j}\left(t\right)+\phi_{j}\left(x_{j}\right)\;\\ y_{j}\left(t\right) & = & x_{1j}\left(t\right)\; \end{array}\right., \end{array}$$

where $x_{j}={\left[x_{1j},x_{2j}\right]}^{T}$ is the state vector for the *j*-th axis. Physically, $x_{1j}$ represents the relative position, and $x_{2j}$ represents the relative velocity. $u_{j}\left(t\right)\in R$ is the control thrust input for the *j*-th axis. $\varphi_{j}\left(\cdot \right)$ and $\phi_{j}\left(\cdot \right)$ represent the unknown control gain function (related to mass) and the lumped nonlinear dynamics (including orbital perturbations and flexible vibrations), respectively. For ease of description, the subscript *j* is omitted in the following derivation. The coordinate transformation is defined as:

(2) $$\begin{array}{c} z_{1}=\frac{e_{h}^{2}e}{e_{h}^{2}-e^{2}} \end{array}$$

(3) $$\begin{array}{c} z_{2}=x_{2}-\alpha_{1}, \end{array}$$

where $e\left(t\right)=y\left(t\right)-y_{r}\left(t\right)$ is the tracking error. The tracking constraint $e_{h}\left(t\right)$ should satisfy $-e_{h}\left(t\right)<e\left(t\right)<e_{h}\left(t\right)$. It should be noted that the prescribed performance function $e_{h}\left(t\right)$ in this paper specifically defines the safety corridor for the servicing spacecraft (the attachment point of the truss). Due to the large moment of inertia of the dragged truss, the towing maneuver is typically performed at low speeds with smooth attitude changes. Under such operating conditions, strictly constraining the tracking error of the spacecraft is the fundamental prerequisite for ensuring the safety of the entire assembly. The scalar bound $e_{h}$ thus represents the maximum allowable deviation of the spacecraft from the planned safe trajectory to prevent the root of the truss from colliding with the surroundings. α is the virtual control law, and $y_{r}\left(t\right)$ is the tracking reference. For Equation (1), the system functions are unknown and cannot be directly used to design the required controller. Therefore, RBFNNs are used to approximate arbitrary unknown nonlinear functions. For a given accuracy τ>0, with a sufficiently large number of nodes *l*, an RBFNN can approximate any continuous function $F\left(Z\right)$ on a compact set $\Omega_{Z}$ such that

(4) $$\begin{array}{c} F_{i}\left(Z\right)={W_{i}}^{T}S_{i}\left(Z\right)+\delta \left(Z\right),\left|\delta \left(Z\right)\right|\leq \tau , \end{array}$$

where $Z\in \Omega_{Z}\subseteq R^{q}$ is the input vector, $W_{i}\in R^{l}$ is the weight vector, l>1 is the number of neural network nodes, $\delta \left(Z\right)$ is the approximation error, and $S_{i}\left(Z\right)={\left[s_{1}\left(Z\right),\cdots ,s_{l}\left(Z\right)\right]}^{T}$ is the basis function vector. $s_{i}\left(Z\right)$ is generally set as a Gaussian function:

(5) $$\begin{array}{c} s_{i}\left(Z\right)=\exp\left[\frac{-{\left(Z-\upsilon_{i}\right)}^{T}\left(Z-\upsilon_{i}\right)}{2\eta^{2}}\right],i=1,2,\ldots ,l, \end{array}$$

where $\nu ={\left[\nu_{i1},\nu_{i2},\cdots ,\nu_{iq}\right]}^{T}$ and η represent the center of the receptive field and the width of the Gaussian function, respectively. $F_{i}\left(Z\right)$ in Equation (4), but this equation is not numbered 32. Assuming it refers to the RBFNN approximation concept) is the dynamic model to be fitted. This work uses three RBFNNs with state-dependent inputs $X_{1}={\left[x_{1},y_{r},{\dot{y}}_{r}\right]}^{T},X_{2}={\left[x_{1},x_{2},y_{r},{\dot{y}}_{r},\lambda ,{\hat{\theta }}_{1}\right]}^{T}$ as input vectors. However, the ideal weight matrix $W_{i}$ is unknown. The adaptive parameter is designed as $\theta_{i}=\left.|W_{i}\right.{|}^{2}$, and its estimate ${\hat{\theta }}_{i}$ is used to compute the driving parameters. $W_{i}$ is an unknown ideal weight vector. Therefore, the adaptive parameter is designed as $\theta_{i}=\left.|W_{i}\right.{|}^{2}$. The first adaptive law is designed as

(6) $$\begin{array}{c} {\dot{\hat{\theta }}}_{1}=\frac{r_{1}}{2l_{1}}{\left(z_{1}\lambda \right)}^{2}S_{1}^{T}\left(X_{1}\right)S_{1}\left(X_{1}\right)-\sigma_{1}{\hat{\theta }}_{1}-\frac{b_{1}{\hat{\theta }}_{1}^{3}}{r_{1}}, \end{array}$$

where $r_{1}$ is a positive design parameter, and let $\lambda =\frac{e_{h}^{2}}{\left(e_{h}^{2}-e^{2}\right)}$. $l_{1}$ is the number of nodes in the neural network. The first virtual control signal is designed as

(7) $$\begin{array}{c} \alpha_{1}=-\frac{z_{1}{\tilde{\alpha }}_{1}^{2}}{{\underline{\varphi }}_{1}\lambda \sqrt{z_{1}^{2}{\tilde{\alpha }}_{1}^{2}+\varepsilon_{1}^{2}}}, \end{array}$$

(8) $$\begin{array}{c} {\tilde{\alpha }}_{1}=K_{11}{\left(\frac{1}{2}\right)}^{\frac{3}{4}}\frac{{\left(z_{1}^{2}\right)}^{\frac{3}{4}}}{z_{1}}+K_{12}{\left(\frac{1}{2}\right)}^{2}z_{1}^{3}+\frac{z_{1}\lambda^{2}}{2l_{1}}{\hat{\theta }}_{1}S_{1}^{T}\left(X_{1}\right)S_{1}\left(X_{1}\right)+\Psi , \end{array}$$

where $K_{11},K_{12}$ are positive parameters, $\varepsilon_{1}$ and $\Psi =\frac{\partial z_{1}}{\partial e_{h}}{\dot{e}}_{h}$ are also positive parameters. The control gains $K_{11},K_{12},K_{21},K_{22}$ play a crucial role in the system’s stability and convergence performance. Specifically, the fractional power terms (related to $K_{11},K_{21}$) dominate the convergence rate when the error is small, ensuring finite-time convergence, while the higher-order terms (related to $K_{12},K_{22}$) handle large initial errors. In practice, these parameters are tuned via a trial-and-error method: starting with small positive values to ensure stability, then gradually increasing them to improve tracking speed until the actuator saturation or noise amplification becomes unacceptable. For the system described by Equation (1), the second virtual control input and adaptive law are designed as

(9) $$\begin{array}{c} {\tilde{\alpha }}_{2}=K_{21}{\left(\frac{1}{2}\right)}^{\frac{3}{4}}\frac{{\left(z_{2}^{2}\right)}^{\frac{3}{4}}}{z_{2}}+K_{22}{\left(\frac{1}{2}\right)}^{2}z_{2}^{3}+\frac{z_{2}}{2l_{2}}{\hat{\theta }}_{2}S_{2}^{T}\left(X_{2}\right)S_{2}\left(X_{2}\right), \end{array}$$

(10) $$\begin{array}{c} {\dot{\hat{\theta }}}_{2}=\frac{r_{2}}{2l_{2}}z_{2}^{2}S_{2}^{T}\left(X_{2}\right)S_{2}\left(X_{2}\right)-\sigma_{2}{\hat{\theta }}_{2}-\frac{b_{2}{\hat{\theta }}_{2}^{3}}{r_{2}}. \end{array}$$

In summary, according to Equation (7), the thruster controller for each degree of freedom can be designed as

(11) $$\begin{array}{c} u_{j}=-\frac{z_{2}{\tilde{\alpha }}_{2}^{2}}{{\underline{\varphi }}_{2}\sqrt{z_{2}^{2}{\tilde{\alpha }}_{2}^{2}+\varepsilon_{2}^{2}}}. \end{array}$$

**Figure 8:**
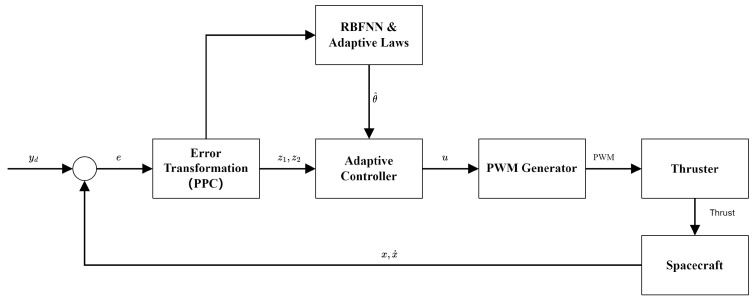
The closed-loop block diagram of the proposed RBFNN-based PPC system.

Furthermore, the motion control system of the spacecraft can be subscribed as Figure 8.

## 4. Stability Analysis

From Equation (1) and the tracking error $e\left(t\right)=y\left(t\right)-y_{r}\left(t\right)$, it can be obtained

(12) $$\begin{array}{cc} \dot{e} & =\dot{y}\left(t\right)-{\dot{y}}_{r}\left(t\right)=\varphi_{1}x_{2}+\phi_{1}-{\dot{y}}_{r}\left(t\right). \end{array}$$

According to Equation (2), one has

(13) $$\begin{array}{c} {\dot{z}}_{1}=\frac{\partial z_{1}}{\partial e_{h}}{\dot{e}}_{h}+\frac{\partial z_{1}}{\partial e}\dot{e}=\Psi +\lambda \dot{e}, \end{array}$$

Consider the Lyapunov function in the following form:

(14) $$\begin{array}{c} V_{1}=\frac{z_{1}^{2}}{2}+\frac{{\tilde{\theta_{1}}}^{2}}{2r_{1}} \end{array}$$

where the design parameter $r_{1}$ is positive, and $\tilde{\theta_{1}}=\theta_{1}-\hat{\theta_{1}}$ is the estimation error of $\theta_{1}$, $\theta_{1}=\left.|W_{1}\right.{|}^{2}$. Furthermore, $z_{2}=x_{2}-\alpha_{1}$, the differentiating of $V_{1}$ gives

(15) $$\begin{array}{cc} {\dot{V}}_{1} & =z_{1}{\dot{z}}_{1}-\frac{{\tilde{\theta }}_{1}{\dot{\hat{\theta }}}_{1}}{r_{1}}=z_{1}\left(\Psi +\lambda \dot{e}\right)-\frac{{\tilde{\theta }}_{1}{\dot{\hat{\theta }}}_{1}}{r_{1}} \end{array}$$

(16) $$\begin{array}{cc} & =z_{1}\left[\Psi +\lambda (\varphi_{1}x_{2}+\phi_{1}-{\dot{y}}_{r})\right]-\frac{{\tilde{\theta }}_{1}{\dot{\hat{\theta }}}_{1}}{r_{1}} \end{array}$$

(17) $$\begin{array}{cc} & =z_{1}\lambda \left(\varphi_{1}z_{2}+\varphi_{1}\alpha_{1}+F_{1}\right)+z_{1}\Psi -\frac{{\left(\lambda z_{1}\right)}^{2}}{2}-\frac{{\tilde{\theta }}_{1}{\dot{\hat{\theta }}}_{1}}{r_{1}}, \end{array}$$

where $F_{1}\left(Z_{1}\right)=\phi_{1}-{\dot{y}}_{r}+\frac{\lambda z_{1}}{2},Z_{1}={\left[x_{1},y_{r},{\dot{y}}_{r},\lambda \right]}^{T}\in \Omega_{Z_{1}}\subseteq R^{4}$ with a compact set $\Omega_{Z_{1}}$. Furthermore, $F_{1}\left(Z_{1}\right)$ contains unknown function $\phi_{1}$, RBFNN is introduced to approximate it over a compact set $\Omega_{Z_{1}}$. Furthermore, it can be obtained as

(18) $$\begin{array}{c} F_{1}\left(Z_{1}\right)=W_{1}^{T}S_{1}\left(Z_{1}\right)+\delta_{1}\left(Z_{1}\right),\;\left|\delta_{1}\left(Z_{1}\right)\right|\leq \tau_{1}, \end{array}$$

where $\tau_{1}>0$.

By applying Young’s inequality, the following can be obtained:

(19) $$\begin{array}{cc} z_{1}\lambda F_{1} & =z_{1}\lambda \left[W_{1}^{T}S_{1}\left(Z_{1}\right)+\delta_{1}\left(Z_{1}\right)\right] \end{array}$$

(20) $$\begin{array}{cc} & \leq |z_{1}\lambda |\left[∥W_{1}∥∥S_{1}\left(Z_{1}\right)∥+\tau_{1}\right] \end{array}$$

(21) $$\begin{array}{cc} & \leq |z_{1}\lambda |\left[∥W_{1}∥∥S_{1}\left(X_{1}\right)∥+\tau_{1}\right] \end{array}$$

(22) $$\begin{array}{cc} & \leq \frac{{\left(z_{1}\lambda \right)}^{2}}{2l_{1}}\theta_{1}S_{1}^{T}\left(X_{1}\right)S_{1}\left(X_{1}\right)+\frac{l_{1}}{2}+\frac{{\left(z_{1}\lambda \right)}^{2}}{2}+\frac{\tau_{1}^{2}}{2}, \end{array}$$

where $X_{1}=\left[x_{1},y_{r},{\dot{y}}_{r}\right]\in \Omega_{X_{1}}\subseteq \Omega_{Z_{1}}$ with a compact set $\Omega_{X_{1}}$, and $l_{1}$ is a positive constant. Furthermore, substituting it into Equation (15) gives

(23) $$\begin{array}{c} {\dot{V}}_{1}\leq z_{1}\lambda \varphi_{1}z_{2}+z_{1}\lambda \varphi_{1}\alpha_{1}+\frac{{\left(z_{1}\lambda \right)}^{2}}{2l_{1}}\theta_{1}S_{1}^{T}\left(X_{1}\right)S_{1}\left(X_{1}\right)+\frac{l_{1}}{2}+\frac{\tau_{1}^{2}}{2}+z_{1}\Psi -\frac{{\tilde{\theta }}_{1}{\dot{\hat{\theta }}}_{1}}{r_{1}}. \end{array}$$

So, the first virtual control signal and the adaptation law are designed as Equations (6)–(8).

As Equation (7), it can be easy to prove that

(24) $$\begin{array}{c} z_{1}\lambda \varphi_{1}\alpha_{1}=-\frac{\varphi_{1}z_{1}^{2}{\tilde{\alpha }}_{1}^{2}}{{\underline{\varphi }}_{1}\sqrt{z_{1}^{2}{\tilde{\alpha }}_{1}^{2}+\varepsilon_{1}^{2}}}\leq -\frac{z_{1}^{2}{\tilde{\alpha }}_{1}^{2}}{\sqrt{z_{1}^{2}{\tilde{\alpha }}_{1}^{2}+\varepsilon_{1}^{2}}}\leq \varepsilon_{1}-z_{1}{\tilde{\alpha }}_{1}. \end{array}$$

For $z_{1}\in R$ and any constant ε>0.

Then, substituting Equations (6)–(8) into Equation (15) results in

(25) $$\begin{array}{c} {\dot{V}}_{1}\leq -K_{11}{\left(\frac{z_{1}^{2}}{2}\right)}^{\frac{3}{4}}-K_{12}{\left(\frac{z_{1}^{2}}{2}\right)}^{2}+\frac{\sigma_{1}}{r_{1}}{\tilde{\theta }}_{1}{\hat{\theta }}_{1}+\frac{b_{1}}{r_{1}^{2}}{\tilde{\theta }}_{1}{\hat{\theta }}_{1}^{3}+\lambda \varphi_{1}z_{1}z_{2}+\rho_{1}, \end{array}$$

where $\rho_{1}=\frac{2\varepsilon_{1}+l_{1}+\tau_{1}^{2}}{2}>0$.

According to Equations (1)–(3), it can be written as

(26) $$\begin{array}{c} {\dot{z}}_{2}={\dot{x}}_{2}-{\dot{\alpha }}_{1}=\varphi_{2}u+\phi_{2}-{\dot{\alpha }}_{1}. \end{array}$$

Choose $V_{2}$ as

(27) $$\begin{array}{c} V_{2}=V_{1}+\frac{z_{2}^{2}}{2}+\frac{{\tilde{\theta }}_{2}^{2}}{2r_{2}}, \end{array}$$

where $r_{2}$ is a positive design parameter and $\tilde{\theta_{2}}=\theta_{2}-\hat{\theta_{2}}$ is the estimation error, $\theta_{2}=\left.|W_{2}\right.{|}^{2}$. Furthermore, obtain

(28) $$\begin{array}{cc} {\dot{V}}_{2} & \leq -K_{11}{\left(\frac{z_{1}^{2}}{2}\right)}^{\frac{3}{4}}-K_{12}{\left(\frac{z_{1}^{2}}{2}\right)}^{2}+\frac{\sigma_{1}}{r_{1}}{\tilde{\theta }}_{1}{\hat{\theta }}_{1}+\frac{b_{1}}{r_{1}^{2}}{\tilde{\theta }}_{1}{\hat{\theta }}_{1}^{3}\\ & \;+\rho_{1}+z_{2}\left(\varphi_{2}u+F_{2}\right)-\frac{z_{2}^{2}}{2}-\frac{{\tilde{\theta }}_{2}{\dot{\hat{\theta }}}_{2}}{r_{2}}, \end{array}$$

where $F_{2}\left(Z_{2}\right)=z_{1}\varphi_{1}+\phi_{2}-\dot{alpha_{1}}+\frac{z_{2}}{2}$, $Z_{2}=\left[x_{1},x_{2},y_{r},{\dot{y}}_{r},{\ddot{y}}_{r},\lambda ,{\hat{\theta }}_{1}\right]\in \Omega_{Z_{2}}\subseteq R^{6}$ with a compact set $\Omega_{Z_{2}}$. For a given $\tau_{2}>0$, a neural network $W_{2}^{T}S_{2}\left(Z_{2}\right)$ is employed to take stock of the unknown function $F_{2}\left(Z_{2}\right)$. Furthermore, $F_{2}\left(Z_{2}\right)=W_{2}^{T}S_{2}\left(Z_{2}\right)+\delta_{2}\left(Z_{2}\right),\left.|\delta_{2}\left(Z_{2}\right)\right.|\leq \tau_{2}$. Using the same method as Equation (22), the following inequality can be written as

(29) $$\begin{array}{c} z_{n}F_{n}\leq \frac{z_{n}^{2}}{2l_{n}}\theta_{n}S_{n}^{T}\left(X_{n}\right)S_{n}\left(X_{n}\right)+\frac{l_{n}}{2}+\frac{z_{n}^{2}}{2}+\frac{\tau_{n}^{2}}{2}, \end{array}$$

where $X_{2}={\left[x_{1},x_{2},y_{r},{\dot{y}}_{r},\lambda ,{\hat{\theta }}_{1}\right]}^{T}\in \Omega_{X_{2}}\subseteq \Omega_{Z_{2}}$ with a compact set $\Omega_{X_{2}}$. Furthermore, $l_{2}>0$ is a design parameter.

As same as Equation (24), it can be written as

(30) $$\begin{array}{c} z_{2}\varphi_{2}u\leq \varepsilon_{2}-z_{n}{\tilde{\alpha }}_{2}. \end{array}$$

Therefore, Equation (28) can be subscribed as

(31) $$\begin{array}{cc} {\hat{V}}_{2}\leq & -K_{11}{\left(\frac{z_{1}^{2}}{2}\right)}^{\frac{3}{4}}-K_{21}{\left(\frac{z_{2}^{2}}{2}\right)}^{\frac{3}{4}}-K_{12}{\left(\frac{z_{1}^{2}}{2}\right)}^{2}-K_{22}{\left(\frac{z_{2}^{2}}{2}\right)}^{2}\\ & +\frac{\sigma_{1}}{r_{1}}{\hat{\theta }}_{1}{\hat{\theta }}_{1}+\frac{\sigma_{2}}{r_{2}}{\hat{\theta }}_{2}{\hat{\theta }}_{2}+\frac{b_{1}}{r_{1}^{2}}{\hat{\theta }}_{1}{\hat{\theta }}_{1}^{3}+\frac{b_{2}}{r_{2}^{2}}{\hat{\theta }}_{2}{\hat{\theta }}_{2}^{3}+\rho_{2}, \end{array}$$

with $\rho_{2}=\frac{2\epsilon_{1}+2\epsilon_{2}+l_{1}+l_{2}+{\tau_{1}}^{2}+{\tau_{2}}^{2}}{2}>0.$

Define $\bar{\mu_{1}}=min\left\{K_{11},K_{21}\right\},\;\bar{\mu_{2}}=min\left\{K_{12},K_{22}\right\}$. By applying a corollary of Cauchy–Schwartz inequality, the formula can be transformed into

(32) $$\left\{\begin{array}{c} -K_{11}{\left(\frac{z_{1}^{2}}{2}\right)}^{\frac{3}{4}}-K_{21}{\left(\frac{z_{2}^{2}}{2}\right)}^{\frac{3}{4}}\leq -{\bar{\mu }}_{1}{\left(\frac{z_{1}^{2}}{2}\right)}^{\frac{3}{4}}-{\bar{\mu }}_{1}{\left(\frac{z_{2}^{2}}{2}\right)}^{\frac{3}{4}}\leq -{\bar{\mu }}_{1}{\left(\frac{z_{1}^{2}}{2}+\frac{z_{2}^{2}}{2}\right)}^{\frac{3}{4}},\\ -K_{12}{\left(\frac{z_{1}^{2}}{2}\right)}^{2}-K_{22}{\left(\frac{z_{2}^{2}}{2}\right)}^{2}\leq -{\bar{\mu }}_{2}{\left(\frac{z_{1}^{2}}{2}\right)}^{2}-{\bar{\mu }}_{2}{\left(\frac{z_{2}^{2}}{2}\right)}^{2}\leq -\frac{{\bar{\mu }}_{2}}{2}{\left(\frac{z_{1}^{2}}{2}+\frac{z_{2}^{2}}{2}\right)}^{2}. \end{array}\right.$$

For $\tilde{\theta_{i}}\hat{\theta_{i}}\leq -\frac{{\tilde{\theta_{i}}}^{2}}{2}+\frac{{\hat{\theta_{i}}}^{2}}{2}$, it can be obtained as

(33) $$\frac{\sigma_{1}}{r_{1}}{\tilde{\theta }}_{1}{\hat{\theta }}_{1}+\frac{\sigma_{2}}{r_{2}}{\tilde{\theta }}_{2}{\hat{\theta }}_{2}\leq -\frac{\sigma_{1}{\tilde{\theta }}_{1}^{2}}{2r_{1}}-\frac{\sigma_{2}{\tilde{\theta }}_{2}^{2}}{2r_{2}}+\frac{\sigma_{1}\theta_{1}^{2}}{2r_{1}}+\frac{\sigma_{2}\theta_{2}^{2}}{2r_{2}}.$$

Substituting Equations (32) and (33) into Equation (31), can get

(34) $$\begin{array}{cc} {\dot{\hat{V}}}_{2}\leq & -{\bar{\mu }}_{1}{\left(\frac{z_{1}^{2}}{2}+\frac{z_{2}^{2}}{2}\right)}^{\frac{3}{4}}-{\left(\frac{\sigma_{1}{\tilde{\theta }}_{1}^{2}}{2r_{1}}+\frac{\sigma_{2}{\tilde{\theta }}_{2}^{2}}{2r_{2}}\right)}^{\frac{3}{4}}-\frac{{\bar{\mu }}_{2}}{2}{\left(\frac{z_{1}^{2}}{2}+\frac{z_{2}^{2}}{2}\right)}^{2}\\ & +{\left(\frac{\sigma_{1}{\tilde{\theta }}_{1}^{2}}{2r_{1}}+\frac{\sigma_{2}{\tilde{\theta }}_{2}^{2}}{2r_{2}}\right)}^{\frac{3}{4}}-\frac{\sigma_{1}{\tilde{\theta }}_{1}^{2}}{2r_{1}}-\frac{\sigma_{2}{\tilde{\theta }}_{2}^{2}}{2r_{2}}+\frac{\sigma_{1}\theta_{1}^{2}}{2r_{1}}+\frac{\sigma_{2}\theta_{2}^{2}}{2r_{2}}+\frac{b_{1}}{r_{1}^{2}}{\tilde{\theta }}_{1}{\hat{\theta }}_{1}^{3}+\frac{b_{2}}{r_{2}^{2}}{\tilde{\theta }}_{2}{\hat{\theta }}_{2}^{3}+\rho_{2}. \end{array}$$

Based on the Young’s inequality as

(35) $${\left|x\right|}^{p}{\left|y\right|}^{q}\leq px+qy.$$

and then, let $x={\left(\frac{3}{4}\right)}^{4},\;y=\frac{\sigma_{1}{\tilde{\theta_{1}}}^{2}}{2r_{1}}+\frac{\sigma_{2}{\tilde{\theta_{2}}}^{2}}{2r_{2}},\;p=\frac{1}{4},\;p_{2}=\frac{3}{4}$, and get

(36) $${\left(\frac{\sigma_{1}{\tilde{\theta }}_{1}^{2}}{2r_{1}}+\frac{\sigma_{2}{\tilde{\theta }}_{2}^{2}}{2r_{2}}\right)}^{\frac{3}{4}}\leq \frac{27}{256}+\frac{\sigma_{1}{\tilde{\theta }}_{1}^{2}}{2r_{1}}+\frac{\sigma_{2}{\tilde{\theta }}_{2}^{2}}{2r_{2}}.$$

Since ${\tilde{\theta }}_{j}{\hat{\theta }}_{j}^{3}={\tilde{\theta }}_{j}\left(\theta_{j}^{3}-3\theta_{j}^{2}{\tilde{\theta }}_{j}+3\theta_{j}{\tilde{\theta }}_{j}^{2}-{\tilde{\theta }}_{j}^{3}\right)$, and combining with Equation (36), then Equation (34) can be obtained

(37) $$\begin{array}{cc} {\dot{V}}_{2} & \leq -{\bar{\mu }}_{1}{\left(\frac{z_{1}^{2}}{2}+\frac{z_{2}^{2}}{2}\right)}^{\frac{3}{4}}-{\left(\frac{\sigma_{1}{\tilde{\theta }}_{1}^{2}}{2r_{1}}+\frac{\sigma_{2}{\tilde{\theta }}_{2}^{2}}{2r_{2}}\right)}^{\frac{3}{4}}-\frac{{\bar{\mu }}_{2}}{2}{\left(\frac{z_{1}^{2}}{2}+\frac{z_{2}^{2}}{2}\right)}^{2}\\ & \;+\frac{3b_{1}{\tilde{\theta }}_{1}^{2}{\hat{\theta }}_{1}^{2}}{r_{1}^{2}}+\frac{3b_{2}{\tilde{\theta }}_{2}^{2}{\hat{\theta }}_{2}^{2}}{r_{2}^{2}}+\frac{b_{1}{\tilde{\theta }}_{1}{\hat{\theta }}_{1}^{3}}{r_{1}^{2}}+\frac{b_{2}{\tilde{\theta }}_{2}{\hat{\theta }}_{2}^{3}}{r_{2}^{2}}\\ & \;-\frac{b_{1}{\tilde{\theta }}_{1}^{4}}{r_{1}^{2}}-\frac{b_{2}{\tilde{\theta }}_{2}^{4}}{r_{2}^{2}}-\frac{3b_{1}{\tilde{\theta }}_{1}^{2}{\hat{\theta }}_{1}^{2}}{r_{1}^{2}}-\frac{3b_{2}{\tilde{\theta }}_{2}^{2}{\hat{\theta }}_{2}^{2}}{r_{2}^{2}}+{\tilde{\rho }}_{2} \end{array}$$

with ${\tilde{\rho }}_{2}=\rho_{2}+\frac{27}{256}+\frac{\sigma_{1}\theta_{1}^{2}}{2r_{1}}+\frac{\sigma_{2}\theta_{2}^{2}}{2r_{2}}$. Similar to Equation (36), Applying Young’s inequality, it can be written as

(38) $$\begin{array}{cc} \frac{3b_{i}{\tilde{\theta_{i}}}^{3}\theta_{i}}{r_{i}^{2}} & \leq \frac{9b_{i}{\tilde{\theta_{i}}}^{\frac{8}{3}}\theta_{i}^{4}}{4r_{i}^{2}}+\frac{3b_{i}\theta_{i}^{4}}{4\kappa^{4}r_{i}^{2}}, \end{array}$$

(39) $$\begin{array}{cc} \frac{b_{i}{\tilde{\theta }}_{i}{\hat{\theta }}_{i}^{3}}{r_{i}^{2}} & \leq \frac{3b_{i}{\tilde{\theta }}_{i}^{2}{\hat{\theta }}_{i}^{2}}{r_{i}^{2}}+\frac{b_{i}\theta_{i}^{4}}{12r_{i}^{2}}, \end{array}$$

where i=1,2, and the design parameter $0<\kappa <{\left(\frac{2}{3}\right)}^{\frac{3}{2}}$. Equation (38) can be obtained by setting $p=\frac{4}{3},\;q=4,\;x=3^{\frac{3}{4}}\kappa {\tilde{\theta_{i}}}^{3},\;y=3^{\frac{1}{4}}\frac{1}{\kappa }\theta_{i}$ and substituting it into Equation (35). For Equation (39), it can be set as $p=2,\;q=2,\;x=\sqrt{6}\tilde{\theta_{i}}\theta_{i},\;y=\frac{1}{\sqrt{6}}\theta_{i}^{2}$.

Substituting Equations (38) and (39) into Equation (37), the following can be obtained:

(40) $$\begin{array}{cc} {\dot{V}}_{2} & \leq -{\bar{\mu }}_{1}{\left(\frac{z_{1}^{2}}{2}+\frac{z_{2}^{2}}{2}\right)}^{\frac{3}{4}}-{\left(\frac{\sigma_{1}{\tilde{\theta }}_{1}^{2}}{2r_{1}}+\frac{\sigma_{2}{\tilde{\theta }}_{2}^{2}}{2r_{2}}\right)}^{\frac{3}{4}}-\frac{{\bar{\mu }}_{2}}{2}{\left(\frac{z_{1}^{2}}{2}+\frac{z_{2}^{2}}{2}\right)}^{2} \end{array}$$

(41) $$\begin{array}{cc} & \;-\left(4b_{1}-9b_{1}\kappa^{\frac{4}{3}}\right){\left(\frac{{\tilde{\theta }}_{1}^{2}}{2r_{1}}\right)}^{2}-\left(4b_{2}-9b_{2}\kappa^{\frac{4}{3}}\right){\left(\frac{{\tilde{\theta }}_{2}^{2}}{2r_{2}}\right)}^{2}+\Xi \end{array}$$

with $\Xi ={\tilde{\rho }}_{2}+\frac{3b_{1}\theta_{1}^{4}}{4\kappa +r_{1}^{2}}+\frac{3b_{2}\theta_{2}^{4}}{4\kappa +r_{2}^{2}}+\frac{b_{1}\theta_{1}^{4}}{12r_{1}^{2}}+\frac{b_{2}\theta_{2}^{4}}{12r_{2}^{2}}$ as a constant.

Let $\hat{\mu_{1}}=\min\left\{\sigma_{1},\sigma_{2}\right\},\;\hat{\mu_{2}}=\left(4-9\kappa^{\frac{4}{3}}\right)\min\left\{b_{1},b_{2}\right\}$, and Equation (40) can be written as

(42) $${\dot{\hat{V}}}_{2}\leq -{\bar{\mu }}_{1}{\left(\frac{z_{1}^{2}}{2}+\frac{z_{2}^{2}}{2}\right)}^{\frac{3}{4}}-{\hat{\mu }}_{1}{\left(\frac{{\tilde{\theta }}_{1}^{2}}{2r_{1}}+\frac{{\tilde{\theta }}_{2}^{2}}{2r_{2}}\right)}^{\frac{3}{4}}-{\hat{\mu }}_{2}{\left(\frac{{\tilde{\theta }}_{1}^{2}}{2r_{1}}+\frac{{\tilde{\theta }}_{2}^{2}}{2r_{2}}\right)}^{2}-\frac{{\bar{\mu }}_{2}}{2}{\left(\frac{z_{1}^{2}}{2}+\frac{z_{2}^{2}}{2}\right)}^{2}+\Xi .$$

And let $\mu_{1}=\min\left\{\bar{\mu_{1}},\hat{\mu_{1}}\right\},\;\mu_{2}=\min\left\{\frac{\bar{\mu_{2}}}{2},\hat{\mu_{2}}\right\}$, then obtain

(43) $${\dot{\hat{V}}}_{2}\leq -\mu_{1}\left\{{\left(\frac{z_{1}^{2}}{2}+\frac{z_{2}^{2}}{2}\right)}^{\frac{3}{4}}+{\left(\frac{{\tilde{\theta }}_{1}^{2}}{2r_{1}}+\frac{{\tilde{\theta }}_{2}^{2}}{2r_{2}}\right)}^{\frac{3}{4}}\right\}-\mu_{2}\left\{{\left(\frac{z_{1}^{2}}{2}+\frac{z_{2}^{2}}{2}\right)}^{2}+{\left(\frac{{\tilde{\theta }}_{1}^{2}}{2r_{1}}+\frac{{\tilde{\theta }}_{2}^{2}}{2r_{2}}\right)}^{2}\right\}+\Xi .$$

According to Equations (14) and (27), using Cauchy–Schwartz inequality and it can be obtained as

(44) $$\begin{array}{cc} V_{2}^{\frac{3}{4}} & \leq {\left(\frac{z_{1}^{2}}{2}+\frac{z_{2}^{2}}{2}\right)}^{\frac{3}{4}}+{\left(\frac{{\tilde{\theta }}_{1}^{2}}{2r_{1}}+\frac{{\tilde{\theta }}_{2}^{2}}{2r_{2}}\right)}^{\frac{3}{4}}, \end{array}$$

(45) $$\begin{array}{cc} V_{2}^{2}={\left(\frac{z_{1}^{2}}{2}+\frac{z_{2}^{2}}{2}+\frac{{\tilde{\theta }}_{1}^{2}}{2r_{1}}+\frac{{\tilde{\theta }}_{2}^{2}}{2r_{2}}\right)}^{2} & \leq 4\left\{{\left(\frac{z_{1}^{2}}{2}+\frac{z_{2}^{2}}{2}\right)}^{2}+{\left(\frac{{\tilde{\theta }}_{1}^{2}}{2r_{1}}+\frac{{\tilde{\theta }}_{2}^{2}}{2r_{2}}\right)}^{2}\right\}. \end{array}$$

and

(46) $${\dot{V}}_{2}\leq -\mu_{1}V_{2}^{\frac{3}{4}}-\frac{\mu_{2}}{4}V_{2}^{2}+\Xi .$$

At this point, the stability analysis of the predefined performance adaptive fixed-time control has been completed.

## 5. Simulation Experiments

This paper verifies the control law of Equation (11) through simulation experiments, using the model of Spacecraft Dynamics from Simulink. The model parameters used in the verification include the spacecraft mass m=300 kg, and the geocentric gravitational constant $GM=39,860,044\times 10^{7}$$m^{3}/s^{2}$. Since the actual spacecraft is planned to operate in a 400 km circular orbit, *r* is taken as a constant r=6,771,393 m, and the orbital angular velocity around the Earth is $\omega_{orbit}=1.13\times 10^{-3}$ rad/s. The unknown force disturbance $z_{F}$ is taken as a normally distributed random number with a mean of 0 and a standard deviation of 0.1 N. Typically, such large unknown forces are not present in space; this larger value is used here to verify the reliability of the orbit control algorithm. It is important to note that these parameters are only used for the simulation model and not for the controller.

The following parameters will be used for the controller. In the simulation, the target trajectories for all three axes are set as

(47) $$\begin{array}{c} \left[\begin{array}{c} y_{d}\left(t\right)=10\sin\left(\frac{\pi }{50}t\right)\\ y_{d}\left(t\right)=10\cos\left(\frac{\pi }{50}t\right)\\ y_{d}\left(t\right)=10\sin\left(\frac{\pi }{50}t\right) \end{array}\right]. \end{array}$$

The error boundaries are all set to $e_{h}=30\exp\left(-\frac{1}{100}t\right)+0.5$. The initial position is $x\left(0\right)={\left[0\;0\;0\right]}^{T}$, and the initial velocity is $\dot{x}\left(0\right)={\left[0\;0\;0\right]}^{T}$. Controller parameters are designed as $K_{11}=1$, $K_{12}=0.001$, $K_{21}=0.01$, $K_{22}=0.1$, $\varepsilon_{1}=\varepsilon_{2}=1$, $r_{1}=r_{2}=\sigma_{1}=\sigma_{2}=b_{2}=0.01$, $b_{1}=1$. These parameters have no detailed impact on the results within the range of ±50%. The initial condition is $\hat{\theta }\left(0\right)={\left[0.2,0.1\right]}^{T}$. Numerical simulations are performed using MATLAB R2021b with a basic time step of 0.1 s. The RBFNN parameters are set as the number of nodes $l_{1}=8$ and $l_{2}=256$, which can ensure that the calculation time of a single time-step controller is less than 0.1 s. To verify the superiority of the proposed algorithm, a PID controller is selected for comparison. The PID controller remains the most widely used strategy in satellite engineering due to its reliability. To ensure a fair comparison, the PID parameters were not chosen arbitrarily but were fine-tuned using the Ziegler-Nichols method to achieve optimal performance for the nominal system. The parameters are set as $K_{p}=\left[0.3399\right]$, $K_{i}=\left[0.01098\right]$, $K_{d}=\left[2.533\right]$.

It is worth noting that we also attempted to conduct ablation studies by implementing the PPC method and the RBFNN adaptive method separately. However, in the scenario with large uncertainties, the “PPC-only” controller failed to maintain the error within the boundary. This led to the error approaching the prescribed limit, causing the error transformation function (Equation (2)) to approach infinity (singularity), resulting in a numerical breakdown of the simulation even with a reduced time step of 0.01 s. Similarly, the “RBFNN-only” approach failed to converge safely during the transient phase. This phenomenon further validates the necessity of coupling RBFNN with PPC: RBFNN reduces the model uncertainty to prevent boundary violation, while PPC guarantees safety during the neural network’s learning process.

**Figure 9:**
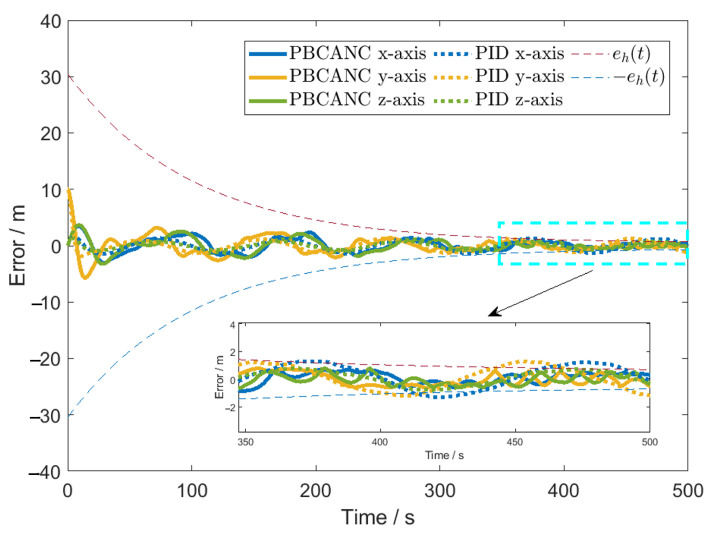
Three-axis tracking error.

The errors for each axis are shown in Figure 9. It can be seen that compared to the traditional PID controller, the proposed controller achieves higher control accuracy while satisfying the prescribed boundary constraints.

## 6. Ground Simulator Verification

**Figure 10:**
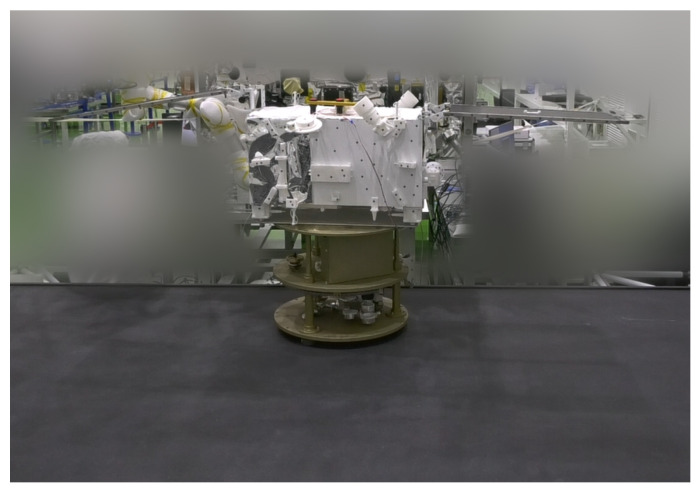
Ground satellite simulator.

In this section, the effectiveness of the proposed control scheme is demonstrated based on the ground satellite simulator shown in Figure 10. The weight and other parameters of the ground simulator used in this experiment are the same as in the simulation. However, the simulator only has two translational axes and one rotational axis, totaling 3 degrees of freedom. To verify the effectiveness of the control method, the proposed algorithm is used to control only the two translational axes of the simulator, while the rotational degree of freedom is set to a fixed orientation. The target trajectories for the two axes are $\left[\begin{array}{c} y_{d}\left(t\right)=\sin\left(\frac{\pi }{285}t\right)\\ y_{d}\left(t\right)=\cos\left(\frac{\pi }{285}t\right)-1 \end{array}\right]$. Limited by the size of the granite platform, the movement range of the ground simulator is restricted to 3 m × 3 m. To demonstrate the effectiveness of the prescribed error boundary of the proposed controller, the error boundary is set to $e_{h}=0.5\exp\left(-\frac{1}{300}t\right)+0.05$. The initial position is $x\left(0\right)={\left[0\;0\;0\right]}^{T}$, the initial velocity is $\dot{x}\left(0\right)={\left[0\;0\;0\right]}^{T}$, and other control parameters are consistent with the simulation settings. The controller used by the ground satellite simulator is developed using Simulink, and the controller operates with a fixed time step of 0.1 s. Pose feedback uses data from the intelligent recognition subsystem.

**Figure 11:**
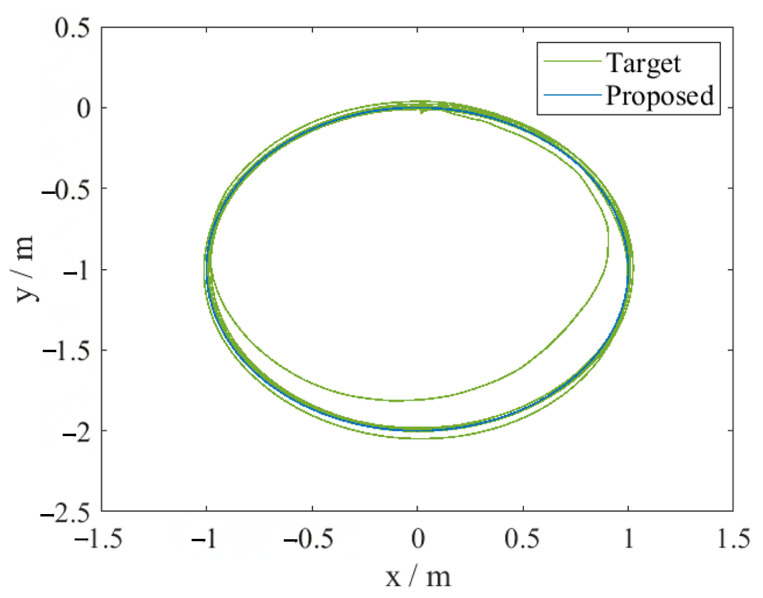
Operating trajectory of the ground satellite simulator.

**Figure 12:**
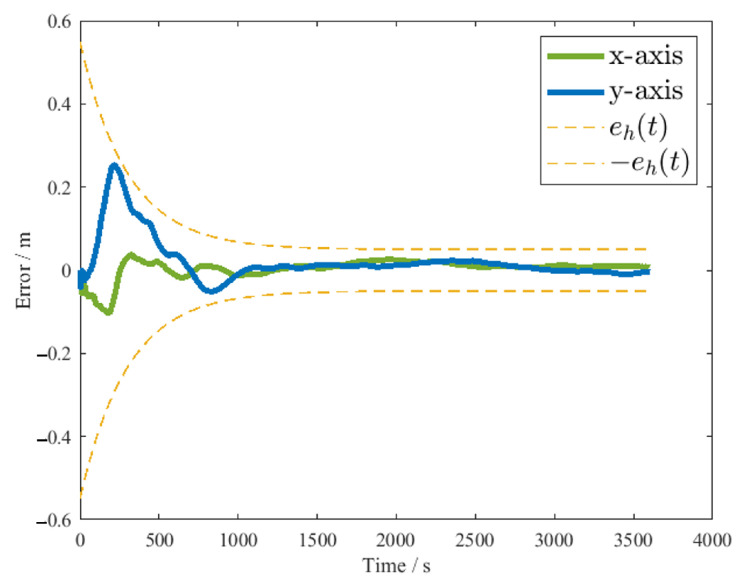
Position tracking error of the ground satellite simulator.

The experimental results are shown in Figure 11 and Figure 12. Figure 11 shows the actual motion trajectory and the ideal trajectory of the ground satellite simulator. It can be observed that the ground satellite simulator deviates significantly from the target trajectory only in the first tracking loop. From the third loop onwards, the actual trajectory basically coincides with the ideal trajectory. Figure 12 shows the tracking errors of the *x*-axis and *y*-axis during the experiment. It can be seen that the errors are controlled within the given error boundaries throughout the entire control process. Before 200 s, the tracking error is relatively large, mainly because the neural network used in this process is continuously approximating the dynamic model of the ground satellite simulator. After 1000 s, the tracking becomes stable, and the errors in both axes can be controlled to about 5 mm, satisfying the given 50 mm error boundary.

## 7. Conclusions

Compared with existing PID schemes, the proposed prescribed-boundary controller can effectively achieve orbit control for servicing spacecraft and possesses the characteristic of a prescribed error boundary. This feature has significant application potential when servicing spacecraft perform close-range proximity operations, allowing the spacecraft to achieve collision avoidance at the controller level while performing proximity maneuvers. Additionally, this controller does not rely on the dynamic model of the spacecraft, which can effectively avoid the problem of difficulty in establishing an accurate dynamic model caused by flexible components such as solar panels on the spacecraft. Furthermore, the RBFNN serves as an effective online estimator, compensating for information that is unmeasurable by traditional physical sensors, thereby closing the loop between sensing and robust execution. Future work will extend the current framework to address the coupled flexible dynamics of the truss more explicitly. Specifically, we aim to investigate the mapping relationship between the spacecraft’s error boundaries and the collision margin of the truss’s distal end. Active vibration suppression and multi-point collision avoidance strategies for the long-span flexible payload will be the focus of our next research phase. Moreover, considering the complexity of assembling ultra-large space infrastructures, we plan to extend the proposed algorithm to multi-satellite cooperative towing scenarios. Future research will focus on developing distributed PPC architectures to address formation-level safety handling and cooperative maneuvering. In such architectures, the robust single-agent controller developed in this paper will serve as the fundamental local control node, while a higher-level consensus algorithm will coordinate the error boundaries of multiple spacecraft to ensure collaborative transport of heavy, large-scale payloads.

## Data Availability

The raw data supporting the conclusions of this article will be made available by the authors on request.
